# Intraperitoneal Chemotherapy for Peritoneal Metastases: Technical Innovations, Preclinical and Clinical Advances and Future Perspectives

**DOI:** 10.3390/biology10030225

**Published:** 2021-03-15

**Authors:** Niki Christou, Clément Auger, Serge Battu, Fabrice Lalloué, Marie-Odile Jauberteau-Marchan, Céline Hervieu, Mireille Verdier, Muriel Mathonnet

**Affiliations:** 1 Service de Chirurgie Digestive, Endocrinienne et Générale, CHU de Limoges, Avenue Martin Luther King, 87042 Limoges CEDEX, France; mathonnet@unilim.fr; 2 Department of Visceral Surgery, Geneva University Hospitals and Medical School, Geneva, Rue Gabrielle Perret-Gentil 4, 1205 Geneva, Switzerland; 3 Laboratoire EA 3842, CAPtuR, Contrôle de l’Activation Cellulaire, Progression Tumorale et Résistance Thérapeutique, Faculté de Médecine, 2 rue du Docteur Marcland, 87025 Limoges CEDEX, France; clement.auger@unilim.fr (C.A.); serge.battu@unilim.fr (S.B.); fabrice.lalloue@unilim.fr (F.L.); jauberte@unilim.fr (M.-O.J.-M.); celine.hervieu@unilim.fr (C.H.); mireille.verdier-sage@unilim.fr (M.V.)

**Keywords:** peritoneal carcinosis, hyperthermic intra-abdominal chemotherapy, complex chemotherapeutic agents, recommendations

## Abstract

**Simple Summary:**

The aim of this review is to emphazise the evolution of intraperitoneal chemotherapy for peritoneal metastasis. Over the last past decade, both delivery modes and conditions concerning hyperthermic intra-abdominal chemotherapy have evolved aiming at improving global and recurrence-free survival of malignant peritoneal diseases. We are waiting now more large randomized controlled trials to demonstrate the efficacy of such treatments.

**Abstract:**

(1) Background: Tumors of the peritoneal serosa are called peritoneal carcinosis. Their origin may be primary by primitive involvement of the peritoneum (peritoneal pseudomyxoma, peritoneal mesothelioma, etc.). This damage to the peritoneum can also be a consequence of the dissipation of cancers—in particular, digestive (stomach, pancreas, colorectal, appendix) and gynecological (ovaries) ones in the form of metastases. The aim of the treatment is a maximal reduction of the macroscopic disease called “cytoreduction” in combination with hyperthermic intra-abdominal chemotherapy to treat residual microscopic lesions. (2) Methods: In this narrative review, we fundamentally synthetize the evolution of this process over time and its impact on clinical applications. (3) Results: Over the last past decade, different evolutions concerning both delivery modes and conditions concerning hyperthermic intra-abdominal chemotherapy have been realized. (4) Conclusion: The final objective of these evolutions is the improvement of the global and recurrence-free survival of primary and secondary malignant peritoneal pathologies. However, more large randomized controlled trials are needed to demonstrate the efficacy of such treatments with the help of molecular biology and genetics.

## 1. Introduction

The peritoneum is a serous membrane covering the intrabdominal cavity. It can be the subject of primary or secondary cancer processes.

Primitive cancers of the peritoneum are rare. They are represented by malignant peritoneal mesothelioma (MPM), primary peritoneal carcinoma, leiomyosarcomas, primary peritoneal serous carcinoma (PPSC), malignant solitary fibrous tumors, desmoplastic small round cells tumor (DSRCT), and peritoneal pseudomyxoma (PMP) (or gelatinous disease of the peritoneum). Their incidence rate is around 6.8 per million [1]. The most frequent type is carcinoma, while MPM is considered as less common but highly aggressive [2]. PMP is estimated at around two cases per million of inhabitants per year. More precisely, PMP is a consequence of the intra-abdominal rupture (evolutive or intraoperative) of what is called the appendicular mucocele, which is a mucinous distention of the appendix lumen originating from an adenoma. PMP is described by the accumulation of mucinous ascites called gelatin on the surface of the visceral and parietal peritoneum. It is important to underline that mucinous cancers from the colon, the pancreas, the urachus, and (more often) the ovary [3] can also extend into the peritoneum as gelatinous ascites.

Secondary tumors of the peritoneal serosa (peritoneal carcinomatosis (PC)) are more common and complicate the course of most intra-abdominal cancers; for colorectal cancers, PC represents the second site of metastasis after liver [4], but it also constitutes potential metastasis localization for gastric [5], pancreatic [6], ovarian [7], and appendicular cancers [8]. Their prognosis is based on the nature of the primary tumor, and their distribution or extension is assessed by the peritoneal cancer index (PCI). Without intervention, the prognosis for PC of any etiology is bleak, with survival of only a few months. PC has long been synonymous with death and has been addressed by palliative care. Today, the aim is to prolong survival and even cure the patient when the disease is not too advanced.

Peritoneal carcinogenesis can be explained by different mechanisms. Serous intrusion and migration, lymphatic or hematogenous dissemination, and finally spontaneous or traumatic (linked to surgeries) dissemination/perforation are those currently recognized in the literature [9]. Tumor cells have to proliferate, survive, and escape from the immune system to finally establish themselves after recirculation and migration. Different cell signaling pathways linked to each step of such metastasis disseminations imply several molecules [10]: (i) tumor shedding and detachment (E-cadherin and epithelial-to-mesenchyme transition (EMT)) [11]; (ii) transport within the peritoneum (actin microfilament system) [11]; (iii) dissemination [12] (intercellular adhesion molecule 1 (ICAM-1), vascular adhesion molecule 1 (VCAM-1), tumor cell receptors like CD44 (cell-surface glycoprotein involved in cell-cell interaction), and cytokines (like tumor necrosis alpha (TNFa), interleukin-1 beta, and interleukin-1 gamma)); (iv) invasion [13] (metalloproteinases and integrins); and (v) proliferation and angiogenesis [14] (epidermal growth factor receptor (EGFR), epidermal growth factor (EGF), tumor growth factor α (TGFα), insulin like growth factor-1 (IGF-1), hypoxia inducible factor (HIF), vascular endothelial growth factor (VEGF), and vascular endothelial growth factor receptor (VEGFR)).

Intraoperative chemoperfusion (IPC), associated or not with prior cytoreduction surgery (CRS), corresponds to a heavy surgical intervention that is scheduled and performed on an open or closed abdomen for peritoneal cancerous involvement. As a curative measure in the majority of operations, this procedure consists of a combination of intra-peritoneal chemotherapies in patients for whom prior cytoreduction has allowed for a sufficient excision (macroscopic unitary tumor remnant). Thus, cytoreduction is evaluated by a radicality score or Completeness Cytoreductive Score (CCS), as described by Sugarbaker [15]. This intraoperative evaluation can be also performed by the “Fagotti score” for ovarian carcinomatosis diffusion and operability, as demonstrated within a prospective study of 113 patients [16]. Usually, it is performed under hyperthermia (hyperthermic intraperitoneal chemotherapy (HIPEC)): Its principle is based on the cytotoxic effect of heat combined with the increased efficacy of certain anti-cancer molecules when they are heated.

The most frequent chemotherapeutics drugs are summarized in Table 1.

The administration of therapeutic agents into the peritoneum is linked to the characteristics of this membrane known to be a transport barrier between the peritoneal cavity and systemic circulation. Mechanistically, it is thanks to the submesothelial blood capillary walls and the surrounding extracellular matrix that the transport is possible. The mesothelial lining may not be implicated because it has been demonstrated in different studies that a peritonectomy does not change IPC pharmakocinetics [21,22,23].

In the last two decades, major innovations in IPC strategy have been realized to improve both its effectiveness and safety. Herein, we performed a narrative review with a specific focus on both the improvement over the last few decades on the technology, pharmacology, and understanding of carcinogenesis mechanisms around IPC while aiming at its efficacy against the aggressiveness of peritoneal carcinomatosis.

## 2. Methods of Delivery

In 1978, Robert Dedrick et al. published theories to deliver IPC for malignant diseases of the peritoneum [24]. Before this date, the peritoneum was considered as a barrier to transport drugs. It was hypothesized that the direct administration of drugs into the abdominal cavity would be able to decrease systemic concentrations of drugs with an increase of local drug concentrations in tumor tissue. This is called the “Dedrick diffusion model” with the concept of dose-intensification thanks to the peritoneal–plasma barrier. During PC, barriers change and evolve. Indeed, the invasion of the peritoneum by malignant tissue leads its partial or complete destruction. Overall, this results in a lack of the mesothelial layer over the tumor, as well as altered vascular and lymphatic microcirculation, which can affect IPC. More precisely, mesothelium loss within the tumor peritoneum is synonymous with not only adhesion reduction and immune system alteration but also rising in macromolecule transport. Concerning lymphatics, within this atmosphere of PC, most of them are obstructed regardless of their localization. All of this provides another mechanism of metastatic diffusion: the systemic route, especially for therapeutic agents with a higher molecular weight than that of albumin. Due to local inflammation and specificities of drugs, capillary permeability increases; moreover, neoangiogenesis linked to the tumor process leads to the formation of new vessels. The vessels have endothelial cells but have lost the characteristics of functional vessels (receptors, muscle tissue, etc.), resulting in modifications of drug distributions. In addition, it is worth noticing that in this context of malignant peritoneum, the interstitium is also modified with pressure changes—the penetration of drugs is thus impacted.

Beyond the pharmacology of IPC, which has specific pharmacokinetic and pharmacodynamic characteristics that need to be considered to overcome these potential diffusion barriers, delivery mode and condition are also of great importance.

Different devices have been used for three decades.

During the first IPC delivery experiment, Speyer and Myers [25] used peritoneal dialysis catheter [26]. Due to an end outside of the body, these catheters were linked to high rates of infections; fully implanted peritoneal access devices (FIPADs) were then developed.

**Figure 1:**
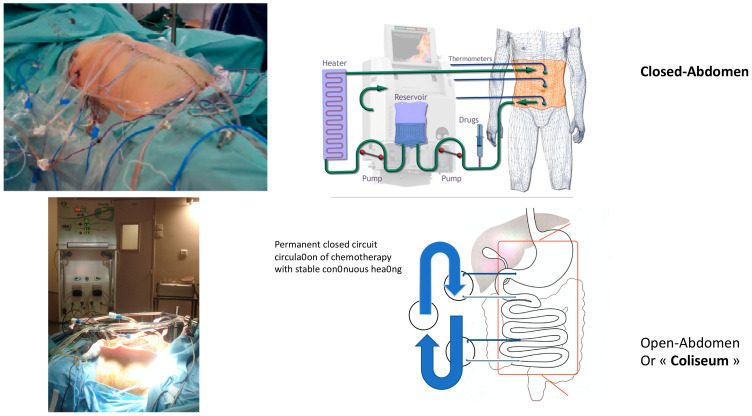
Hyperthermic intraperitoneal chemotherapy (HIPEC).

It is worth noticing that IPC can be performed with either an “open abdomen,” called a “coliseum,” or a “closed abdomen” (Figure 1).

While the open technique is more frequent in European and Asian Centers, the closed one is mainly popular in the USA.

The open method, as described well by Sugarbaker in 2005 [27], argues the possibility to follow the process with direct intraabdominal visualization and distribution harmonization. Nevertheless, there are some difficulties in maintaining constant hyperthermia. On the other hand, with the major inconvenience of not being able to see the abdominal cavity, the closed technique avoids the risk of cytotoxic contamination by the staff in the theatre. Furthermore, it leads to stable high temperature and pressure, positively impacting tissue penetration. A recent retrospective study of patients operated on cytoreductive surgery with HIPEC between 2000 and 2017 from the United States HIPEC collaborative database showed no difference in terms of post-operative complications, cancer recurrences, and overall survival outcomes [28].

In the last decade, different modalities of IPC delivery have been implemented in order to improve its efficacity.

### 2.1. Delivery Conditions

#### 2.1.1. Hyperthermia = Intraoperative Chemoperfusion under Hyperthermic Conditions (HIPEC)

Intraoperative chemoperfusion is usually performed under hyperthermic conditions (HIPEC) [29]. This condition leads to an enhancement of the penetration of cytostatic drugs into tumor tissue, as well as synergy between them. Moreover, the hyperthermia itself provokes an increase of lysosome numbers in malignant cells, thus leading to an increase death rate of these cells [30]. High levels temperature between 41 and 43 °C also induce decreases of blood flow in tumors cells in combination with an inhibition of oxidative metabolism without anaerobic glycolysis modifications. Overall, these phenomena induce a low pH with increased numbers of lysosome-generating cancer cells [31].

The impact of hyperthermia in association with chemotherapeutic agents seems to begin at 39 °C and steadily increases with the temperature. In vivo, on rat models, it was demonstrated that the temperature cannot be higher than 44 °C [32]. Beyond this figure, the bowel can be burnt and anastomotic leakage can occur. Clinical studies have compared IPC in conditions of hyperthermia to those of normothermia. The recent Belgian study of Gremonprez et al. did not show any differences in terms of morbidity, anastomotic leakages rates, and mortality between the two conditions in the context of oxaliplatin intraperitoneal injection.

#### 2.1.2. Pressurized Intraperitoneal Aerosol Chemotherapy (PIPAC)

IP drug delivery can be performed under the form of pressurized intraperitoneal aerosol chemotherapy (PIPAC) during laparoscopy [33,34,35,36]. The cytotoxic solution is injected under a maximal pressure of 20 bar, and the resulting aerosol is dispersed in the abdomen [37]. This innovative system tries to overcome the IPC boundaries of limited cytotoxic direct penetration into tumoral tissue and unequal drug distribution into the peritoneum [24]. PIPAC has the advantage of a higher pressure that does not exceed tumor interstitial pressure. However, it seems important to underline that the majority of the studies investigating this procedure since its introduction in 2011 have not encompassed the IDEAL (Idea, Development, Evaluation, Assessment, Long-term study, Framework) framework [38]. The PIPAC procedure was meticulously tested in a porcine model and compared to washing techniques. These experiments demonstrated the peritoneal distribution of chemotherapy, as well as superior tissue penetration [39]. The clinical application of PIPAC was developed by Professor Reymond’s group at the Marien-Krankenhaus in Herne, Germany, in 2011; in this context of human clinical studies, systemic absorption, hepatic toxicity, and renal toxicity were insignificant [40,41].

Thanks to the physical properties of both gas and pressure, PIPAC has shown amelioration in drug penetration. The hypothesis is that adding an electrostatic field can lead to an enhancement of charged droplet precipitation as well as tissue penetration, creating anticancer efficacy amelioration (electrostatic PIPAC: “ePIPAC”). A recent study focusing on patients with unresectable carcinomatosis showed feasibility and preliminary efficacy [42]. Clinical trials are in progress, and the first results of a Dutch one (NCT03246321) concerning unresectable colorectal carcinomatosis underlined comparable results between ePIPAC and systemic chemotherapy [43]. However, it encompassed 20 patients and this study calls for more prospective research.

Regarding PIPAC itself, a recent meta-analysis demonstrated both its safety and its anti-tumoral activity on peritoneal carcinomatosis [44]. Studies such as the randomized controlled trial PARROT (NCT02735928) that are aimed at proving the therapeutic efficacy of PIPAC for recurrent resistance ovarian cancer are eagerly awaited [45].

#### 2.1.3. Specific Delivery Conditions

##### Normothermia

The condition of normothermia can be performed during NIPS (neoadjuvant intraperitoneal chemotherapy in association with systemic chemotherapy perfusion) and EPIC (early postoperative intraperitoneal chemotherapy) but also during long-term postoperative IPC. Different clinical studies have shown the efficacy of EPIC [46] and long term postoperative IPC [47] in such a condition of normothermia.

##### Laparoscopic HIPEC

Practically, laparoscopic HIPEC corresponds to a kind of “closed” perfusion where inflow and outflow catheters are inserted through the port sites.

This methodology has different aims. Firstly, before cytoreduction, it aims to evaluate the burden of PC. Secondly, in a curative way, it aims to perform CRS and HIPEC for patients with limited PC [48]. Finally, it can lead to palliative [49] or prophylactic [50] HIPEC.

### 2.2. Delivery Modes

#### 2.2.1. Delivery Mode of IPC: Time Factor

In the last few decades, modalities of administration have increasingly modified. Metronomic dosing with the continuous administration of low levels of conventional chemotherapy [51] has recently attracted interest because it avoids long breaks, thus leading to continuous exposure to the drug.

**Figure 2:**
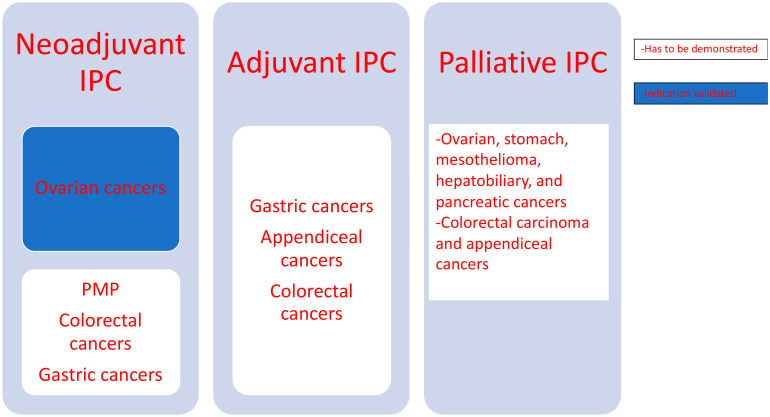
Different indications neoadjuvant, adjuvant and palliative of intraperitoneal chemotherapy. PMP: peritoneal pseudomyxoma.

The most common perioperative administration of IPC is HIPEC. Nevertheless, in daily clinical routine, IPC can be initiated according to various time schedules with different effects (Figure 2).

a. Neoadjuvant IPC

With the help of systemic chemotherapeutic agents, neoadjuvant IPC aims to make surgery easier by trying to reduce IP disease (“to reduce the visible tumor burden”), with a reduction of the PCI and the eradication of micrometastasis and detached cancer-cells before surgical cytoreduction [52]. More precisely, different publications have defined it with a higher pathological response and complete cytoreduction leading to improved-survival. Furthermore, it seems to encompass few toxicities, especially of grades III–V [53]. Thus, this management has been described for gastric and ovarian cancers with both clinical and radiological follow-ups [54,55]. Two Japanese prospective studies using, respectively, IP docetaxel or mitomycin C (MMC) and cisplatin (CDDP) demonstrated that more than half of the patients had histologic responses with an improved overall survival (OS) [56,57]. Regarding ovarian cancers, in 2011, Muñoz-Casares et al. [58] showed a decrease in both PCI and CA-125 (carbohydrate antigen 125) levels by using paclitaxel in an IP neoadjuvant setting with 10 patients having stage IIIc ovarian cancer. For PMP, the use of neoadjuvant intravenous chemotherapy did not impact on both OS and disease free-survival (DFS) in different studies. However, its use within the peritoneum was recently studied by Prabhu et al. in 2020 [59]. They demonstrated a few complications and low mortality rates. Moreover, there were high rates of complete cytoreduction in association with tumor regression. Further studies are now needed in order to assess the impact of neoadjuvant IPC on survival.

Recently, a new bidirectional intraperitoneal and systemic induction chemotherapy (BISIC) was described [60]. This consists of the administration of drugs from both sides of the peritoneal surface, thus allowing for the treatment of a larger area of sub-peritoneal tissue than IP injection alone. Results concerning both OS and tumor response were favorable, with few toxicities [53].

While the main advantage of neoadjuvant IPC is to reduce IP burden, some drawbacks such as fibrosis, adhesions, and more morbidity at the time of cytoreduction can appear [61]. Consequently, prospective studies are required to certify the effectiveness and safety of such a procedure. Several clinical trials are recruiting, especially in colorectal cancers (NCT03253133) and gastric cancers (NCT04308837).

Overall, it appears that laparoscopic preoperative PCI evaluation and cytology assessment after Neoadjuvant IPC are mandatory in order to select patients for CRS.

b. IPC, Extensive Intraoperative Peritoneal Lavage (EIPL), and HIPEC

IPC is the most frequent method, and it is performed immediately after the cytoreduction during the same intervention. It consists of the direct administration of chemotherapeutic agents inside the peritoneum (either with the “open” or “closed “abdomen). It can be associated with the simultaneous intravenous administration of chemotherapy or immediately before HIPEC (maximum of 60 min prior).

In 2009, Kuramoto et al. described another protocol called extensive intraoperative peritoneal lavage (EIPL) [62], where they extensively performed a peritoneal lavage with chemotherapeutic drugs. The aim of this lavage is to remove floating cancer cells from the peritoneum, as well as the blood and lymphatic vessels, after CRS. In this randomized controlled trial (RCT), they demonstrated an enhanced OS after CRS combined with EIPL compared to CRS alone or intraoperative IPC plus CRS.

Furthermore, as hyperthermia higher than 42 °C increases drug cytotoxic effects and enhances the depth of chemotherapeutic agents’ penetration into the peritoneal cavity, CRS in association with HIPEC has been tested in different studies for different origin types of PM.

A main advantage in terms of survival has been highlighted in RCTs with the use of CRS and HIPEC for both the prevention [63,64] and treatment [65,66] of PC in some cancers such as gastric and colorectal. However, HIPEC by itself may reinforce morbidity, and complications are considered as an independent prognostic factor of OS [67]. Thus, a 2007 meta-analysis of Yan et al. [68] regarding CRS/HIPEC for gastric cancer underlined increased neutropenia and abdominal abscess after CRS plus HIPEC in comparison to CRS alone. The more recent 2020 multicentric retrospective study of Gamboa et al. [69] for patients having undergone CRS/HIPEC for appendiceal/colorectal cancer demonstrated that higher rates of complications are linked to infections, which themselves lead to decreased OS.

Beyond the use of HIPEC, intraperitoneal chemotherapy by itself, without hyperthermia, may lead to some complications, especially in the context of surgery with bowel resections and sutures (where fecal leaks can occur). Recently, a meta-analysis focusing on this topic provided some evidence about increasing risk of anastomotic leaks after colorectal cancer surgery with normothermia IPC [70].

c. Early Postoperative Intraperitoneal Chemotherapy (EPIC)

While HIPEC is performed immediately after CRS in theatres for around less 120 min, EPIC is administered post-operatively. More precisely, three or five days following CRS, associated with HIPEC or not, drains are left in the abdomen to infuse chemotherapy into the abdominal cavity for around 23 h a day for 5–7 days [18].

After 23 h of infusion, there is a drainage for 1 h prior to each re-administration. The cytotoxic drugs used target usually the cell cycle. This implies that longer periods of cell contact with the drugs to lead to cell death. The main aim of EPIC is to eradicate micrometastasis within the peritoneum, as well as floating cancer cells called “peritoneal free cancer cells (PFCCs)”.

The literature is conflicting regarding advantages and drawbacks. For example, some retrospective studies focusing on colorectal and appendiceal cancers with PC have demonstrated both safety and efficacy in contrast to others having underlined increased postoperative morbidity and uncertain additional benefit on OS [71]. Similarly, these contradictious results were found for PC with gastric cancers [72,73].

#### 2.2.2. Delivery Mode of IPC: Carrier Drug Factor

Different carrier solutions with varied tonicity and molecular weight have been developed for the majority of chemotherapies used. By modifying these two parameters according to chemotherapy type, the exposure of IP cancer cells to chemotherapy and drug availability in the peritoneal cavity can be changed and chosen.

Another carrier deliver, thermosensitive hydrogels—naturally derived, synthetic, or mixed—represent liquid solutions at room temperature that turn into gels at body temperature, leading to increased exposure [74]. More precisely, hydrogels are three-dimensional networks made up of polymers in which hydrophilic domains or groups are present in an aqueous environment. They allow for the controlled release of drugs. There are multiple advantages of these extended-release systems [75]. Indeed, they allow for prolonged maintenance of the therapeutic concentration of the drug, a reduction in undesirable side effects by targeting the drug to a cell type, a reduction in the dose required for the therapeutic action, better tolerance by patients of the dosage, and a suitable tool for the administration of biopharmaceuticals with a short half-life in vivo (proteins and peptides). In addition, the hydrogel formulation makes it possible to consider the distribution of several active molecules simultaneously in order to increase therapeutic efficacy by the additivity of effect or synergy.

Recently, nanotechnology has demonstrated potential for nanoparticles (particles smaller than 100 nm) to be good drug carriers. For PC, several nanocarrier conjugates with chemotherapy, immunotherapeutic agents, and antibodies are under investigation; for example, Abraxane^®^ (a nanoparticle albumin-bound paclitaxel) is going to be analyzed in a phase I clinical trial (NCT00825201).

To enhance particle interactions with the peritoneum, the conventional chemo-aerosols that PIPAC tends to use now have nanoparticle agglomeration. While PIPAC uses an intracavitary aerosol generator (IAG), intraperitoneal nano aerosol therapy (INAT) needs an extra-cavitary aerosol generator (EAG) to create smaller particles. More precisely, thanks to an airstream travel, aerosols have time to crystallize. These nano-crystallized chemotherapies lead to higher tissue penetration rates that surpass that of PIPAC [76].

Different limitations of both the technique and its prognosis concerning these delivery modes have been described [77]. Consequently, a new tool recently emerged: foam-based intraperitoneal chemotherapy (FBIC) [78]. The first results in ex vivo models showed its feasibility. It seems encouraging, but further studies are needed to demonstrate its efficacy.

## 3. Evolution of the Drugs Used: From Unsophisticated Intra-Abdominal Chemotherapy to Complex Systems

### 3.1. A General Overview

There are both many pharmacodynamic and pharmacokinetic factors that can influence the effects of the IP chemotherapeutic agents. Pharmacokinetic variables such as the molecular weight of the drug, but also its dosage, its hepatic metabolism and renal clearance, the volume of the carrier solution, the kind of carrier solution, open or closed abdominal lavage, intra-abdominal pressure, the duration of IPC, the extension of the peritoneum resection, pharmacodynamic variables defined as tumor nodule size, the tumor density, extracellular matrix, vascularity, and the temperature can impact IPC efficacy.

Moreover, these characteristics are not sufficient to determine a perfect algorithm of IPC administration to be effective against a tumor. Tumor heterogeneity needs to be taken into account to find the adequate chemotherapeutic agent: the best sensitivity of monochemotherapy or combined chemotherapies with respect to the tumor profile in question without adding morbidities linked to the IPC regimen itself is essential. “A patient-tailored” drug choice thus has to be performed [79,80]. Predictive biomarkers that reflect the tumor biology are consequently needed to determine in order to conduct the optimal management of IPC.

### 3.2. Contribution of Current Basic Research—Perspectives

Recently, many in vivo studies on animals (mice) have been implemented, highlighting the possibility to adapt the IP chemotherapeutics to be more sensitive to different types of gynecologic and digestive cancers. Different mechanisms of resistance, such as epithelial–mesenchymal transition, autophagy, and exosomes, are targeted.

For instance, despite cisplatin being a commonly used drug to treat gastric cancers, it presents a protective autophagy that leads to a decrease in chemosensitivity of gastric cells. This has been demonstrated by the fact that cisplatin is able to inhibit O-6-methylguanine-DNA methyltransferase (MGMT), which is a suicide DNA damage repair enzyme and increase the expression of the autophagy-related gene ATG4B [81]. Similarly, in gastric cancers, phosphoglycerate kinase 1 (PGK1), a glycolytic enzyme that plays role in autophagy and tumorigenesis [82], has been studied thanks to solid tumors coming from a human gastric cancer cell line, MKN45, established from the poorly differentiated adenocarcinoma of the stomach (medullary type) of a 62-year-old woman, grown in a orthoptic xenograft nude mice model and then grafted into the gastric subserosa of other nude mice—the inhibition of PGK1 by short hairpin RNA (shRNA) induced an increased IP cytotoxicity of 5-fluorouracil (5-FU) in contrast to 5-FU alone in gastric cancer with peritoneal metastasis [83].

In colorectal cancer, 5-FU plays a key role as a chemotherapeutic agent. It is well known that p53 status can modify sensitivity to 5FU; when *TP53 (Tumor Protein 53)* is mutated, 5-FU chemoresistance increases [84]. In 2020, Zhan et al. demonstrated that β elemene (1-methyl-1-vinyl-2,4-diisopropenyl-cyclohexane), a natural product present in several plants, can reverse the resistance of the colorectal cancer cell line HCT116, which is deficient for p53 (*TP 53* mutated). More precisely, thanks to a HCT116p53^−/−^ xenograft model, intraperitoneal injections of β elemene plus 5-FU induced tumor volume inhibition, thus demonstrating thus reversion of the resistance of HCT116p53^−/−^ to 5-FU [85].

Recently, peritoneally disseminated metastases of gastric, ovarian, and pancreas cancers have been treated with a DFP-10825 formulation [a cationic liposome and short-hairpin RNAi molecule for thymidylate synthase (TS shRNA)] and injected intraperitoneally [86]. This formulation corresponds to an adenoviral vector-expressing synthesized shRNA that alters the target protein Wnt2B (Ad-shWnt2b) to thymidylate synthase (TS). Wnt2B is a signaling protein belonging to the Wnt signaling pathway regulated by Snail expression linked to the EMT.

Overall, beyond the adaptation of treatments is a question of highlighting diagnostic markers of PC and its aggressive characteristics [87]. Personalized therapeutics are going to be implemented such as the clinical trial Oncogramme (NCT03133273) that our University Hospital of Limoges has initiated.

Beyond the use of chemotherapeutic agents, novel biological cancer therapies like immunotherapy or oncolytic virotherapy can be integrated. Indeed, in the last two decades, basic fundamental research has outlined the importance of immune check-points in cancerology [88]. These are receptors that intervene in the modulation of the activation of immune cells in order to limit the duration and intensity of the immune reaction. Cancer cells are able to hijack this checkpoint system to their advantage. Currently, the anti-checkpoint drugs used in oncology target inhibitory receptors such as CTLA4 (cytotoxic T lymphocyte-associated antigen 4) or PD1 (programmed cell death protein 1) and its PD-L1 ligand. Antitumor virotherapy (also called oncolytic immunotherapy), on the other hand, involves the specific infection of tumor cells with a virus to kill them. Not only are cells killed by the virus but also the resulting cellular waste stimulates the immune system against the tumor [89].

Concerning immunotherapy, clinical studies with immune-modulating agents (NCT02219893), monoclonal antibodies (mAbs) (NCT01099644), chimeric antigen receptor T cells (CAR-T cells) [90], and immune checkpoint inhibitors (ICIs) (NCT03508570) are being performed. They are showing promise for the disease control and the induction of long-lasting antitumor immunity. The rationale behind immunotherapy being used in the treatment of PC comes from the specific characteristics of the peritoneum, which includes CD8+ T cells, CD4+ T cells, and the secretion of proinflammatory cytokines (such as interleukin-1, interleukin-6, prostaglandin E2, interleukin-2, and interferon-gamma).

Different oncolytic viruses selectively bind cancer cells, thus inducing tumor death. They are the targets of several current clinical trials, e.g., the clinical trial NCT03663712 is a phase 1 clinical study that consists of intra peritoneal virotherapy with talimogene laherparepvec (T-VEC) (herpes simplex virus) after prior vaccination for peritoneal surface dissemination from gastrointestinal or recurrent, platinum-resistant ovarian cancer. However, like other therapies, oncolytic virotherapy has a wide range of barriers including elimination by antibodies (after vaccination in childhood and after first virotherapy) or T cells [91] and inactivation by unspecific hemagglutination or by components of the complement system [92].

Recent approaches combining these novel biological cancer therapies to standard ones such as chemotherapies and radiation have the ultimate aim to remove all the barriers that have been preventing them from complete effectiveness.

## 4. Evolution of the Clinical Indications of HIPEC

### 4.1. Recognized Indications

In combination with CRS, HIPEC is recognized as necessary for treating different conditions [93] (Table 2) like ovarian cancer following neoadjuvant chemotherapy, peritoneal mesothelioma (PM), PMP, and PC from different cancerous origins and without extra-abdominal metastases [adenocarcinoma of the appendix or goblet cell carcinoma of the colon, rectum, or small bowel (SB)].

Firstly, regarding ovarian cancers, a recent Dutch randomized controlled trial demonstrated the benefits of the addition of HIPEC to interval cytoreductive surgery for patients with stage III epithelial ovarian cancer in terms of recurrence-free survival and overall survival without higher rates of side effects (NCT00426257) [94] despite some debates [95]. Furthermore, both the National Comprehensive Cancer Network^®^ (NCCN) (NCCN clinical practice guidelines Version 1.2019–8 March 2019 OV-2) and the French society of gynecological and oncological surgery experts with the INCA (National Institute of the Cancer, Institut National du Cancer) label recommend HIPEC with cisplatin at the time of interval debulking surgery following neoadjuvant chemotherapy for stage III ovarian disease (FIGO [International Federation of Gynecology and Obstetrics] III).

Furthermore, different studies have emphasized HIPEC as a potential tool in the treatment of recurrent and upfront surgery for ovarian cancer thanks to the gain of survival [96,97,98,99,100].

Concerning PM and PMP, which are rare entities, no randomized controlled trials are found in the literature.

PM constitutes around a third of all the mesotheliomas. With a poor prognosis and a well-known intrinsic chemoresistance, different studies have demonstrated the positive impact of CRS with HIPEC for patients with a resected disease [101], thus leading to a survival benefit.

PMP is a clinical syndrome involving the development of mucin in the peritoneum due to mucinous neoplasia that originate from the appendix in the majority of cases [102]. The Peritoneal Surface Oncology Group International (PSOGI) came to a consensus in 2016 to differentiate PMP and associated appendiceal neoplasms with the following classification:

- Low-grade appendiceal mucinous neoplasm (LAMN).
- High-grade appendiceal mucinous neoplasm (HAMN).
- Mucinous adenocarcinoma.
- Poorly differentiated mucinous adenocarcinoma with signet ring cells.
- Signet ring carcinoma.

In parallel, the peritoneal disease component of PMP was also redefined with different sub-groups:

- Acellular mucin.
- Low-grade mucinous carcinoma peritonei or disseminated peritoneal adenomucinosis (DPAM).
- High-grade mucinous carcinoma peritonei or peritoneal mucinous carcinomatosis (PMCA).
- High-grade mucinous carcinoma peritonei with signet ring cells or peritoneal mucinous carcinomatosis with signet ring cells (PMCA-S).

Retrospective series in the current literature have shown the safety and effectiveness of CRS combined with HIPEC in comparison with standard treatments. Recently, a prospective study from a unique Italian center collected data from 32 patients with appendiceal origin PMP between 2008 and 2016 [103] confirmed this. Thanks to CRS associated with closed-abdomen HIPEC, this procedure as found to result in a five-year overall survival rate of 58%.

To date, many retrospective studies have shown some benefits of patients with colorectal cancers and isolated PC. Various RCTs are recruiting (NCT02179489, NCT04370925…); however, as HIPEC implies significant morbidity and mortality, with heterogeneous results concerning its efficiency in this type of cancers, the NCCN has edited guidelines for colon cancer that state that complete CRS and/or IPC is possible in experienced centers for selected patients, that is to say with limited PC for whom the complete removal of all known tumor can be achieved (R0). Recently, the RCT called “PROPHYLOCHIP” (NTC-01226394) did not demonstrate any improvement in survival for patients with high risk of PC [66].

Concerning SB cancers and PC, a Dutch retrospective study from the Netherlands Cancer Registry including 1428 patients with SB adenocarcinoma, of whom 13% (s181) had PC, demonstrated a higher survival when patients were treated with CRS and HIPEC.

### 4.2. Indications in Evaluation

Limited PC resulting from gastric cancers seems sensitive to complete CRS. However, there are no current recommendations for this management without an RCT. Several studies have demonstrated its efficacy in Asian population. The following two Western RCTs are trying to bring answers for Caucasian patients: “GASTRICHIP” (NCT01882933) and “GASTRIPEC” (NCT02158988).

PIPAC is used as a palliative treatment in the frame of PC evolution after different lines of systemic chemotherapies. While the extent of PC is usually determined by the PCI, it is difficult to make the same during PIPAC. Indeed, active PC versus inactive PC is rather macroscopically indistinguishable. Consequently, it is the histological analysis of biopsies before PIPAC with the determination of the “Peritoneal Regression Grading System (PRGS),” which is the most relevant. This system is based on a global analysis of two components: tumor cells and regression features. Both the tolerance and safety of this procedure seem reached [104]. Despite encouraging results with increased survival after PIPAC [105,106], its efficacy and, thus, its specific indications need to be underlined thanks to a robust methodology such as future randomized controlled trials. The procedure is very standardized as depicted and follows very strict safety guidelines. Currently, three applications are recommended in a three-month period. The currently recommended chemotherapy regimens are: 92 mg/m^2^ of oxaliplatin for colorectal carcinoma and appendiceal cancers and 1.5 mg/m^2^ of doxorubicin plus 7.5 mg/m^2^ of cisplatin for other etiologies like ovarian, stomach, mesothelioma, hepatobiliary, and pancreatic tumors. Taxane use is under investigation in a phase I–II trial for gastrointestinal and ovarian peritoneal metastases [107]. The use of these different drugs was established in a recent multicenter study [108]. It is essential that all indications for PIPAC be discussed and confirmed at a multidisciplinary oncology conference, and patients should preferably be treated in clinical studies. Recently, two RCTs were launched to demonstrate positive impact on overall survival after PIPAC: for patients with unresectable MPM, NCT03574493 [109], and for those with PM from gastric cancer (PCI > 8), NCT04065139 [110].

## 5. Conclusions

PC is considered to be a disease with poor prognosis. Historically, surgical treatment was not integrated into the management care. Since the 1990s, CRS in association with HIPEC has emerged as a promising treatment for PC linked to ovarian or gastrointestinal tumors without extra-abdominal metastasis. Despite the evidence of significant morbidity and mortality, different recent studies have continued to demonstrate the positive impact of this management by ameliorating the overall survival of patients. However, other studies in the literature have questioned the legitimacy of this procedure. Thanks to technological advances in IPC’s different delivery modes and conditions over the past decade, in combination with future sensitivity predictive biomarkers that will be highlighted (leading to appropriate patient selection in accordance to tumor biology), IPC will probably be a commonly used treatment because it combines efficacy and safety for precise indications in the years to come.

## Figures and Tables

**Table 1:** Most commonly chemotherapeutic agents used during intraoperative chemoperfusion (IPC) for peritoneal carcinomatosis (PC). HIPEC: hyperthermic intraperitoneal chemotherapy.

Drug Class	Subgroup	Drug	AUC (Area under the Curve) Ratio	Synergism with Heat	with Normothermia/with HIPEC	Indications
Alkylating agents	Platinum agents	Cisplatin	7	Yes	+/+ [17,18]	Ovarian cancers
		Carboplatin	18			Bladder cancers
		Oxaliplatin	16			Colorectal cancers
						Gastric cancers
Topoisomerase inhibitors	Topoisomerase II inhibitors	Anthracyclines (Doxorubicin)	230	Yes	−/+ [19]	Gastric cancers
		Mitoxantrone	15.2			
Antimetabolites	Pyrimidine antagonists	5-Fluorouracil (5-FU)	250	Minimum	+/− [18]	Urothelial cell carcinoma, Colorectal cancer
						Pancreatic cancer
Mitotic inhibitors	Taxanes	Docetaxel	1000	No	+/− [20]	Ovarian and gastric cancers
		Paclitaxel				
Antibiotic	Mitomycin C		23.5	Yes	−/+ [18]	Bladder carcinoma
						Colorectal cancers
						Appendiceal mucocele

**Table 2:** Summary table of curative indications of HIPEC.

Recognized Indications	Indications under Evaluation
Stage III epithelial ovarian cancer	Peritoneal carcinomatosis (PC) from gastric cancers without extra-abdominal metastases
Peritoneal mesothelioma (PM)	Peritoneal carcinomatosis (PC) from colorectal cancers without extra-abdominal metastases
Pseudomyxoma peritonei (PMP)	
Peritoneal carcinomatosis (PC) from different cancerous origins, without extra-abdominal metastases: adenocarcinoma of the appendix, goblet cell carcinoma of the small bowel (SB)	

## Data Availability

Not applicable.
